# Levodopa–carbidopa intestinal gel in advanced Parkinson’s disease: long-term results from COSMOS

**DOI:** 10.1007/s00415-023-11615-3

**Published:** 2023-02-18

**Authors:** Alfonso Fasano, Rocío García-Ramos, Tanya Gurevich, Robert Jech, Lars Bergmann, Olga Sanchez-Soliño, Juan Carlos Parra, Mihaela Simu

**Affiliations:** 1 Edmond J Safra Program in Parkinson’s Disease and the Morton and Gloria Shulman Movement Disorders Clinic, Toronto Western Hospital-UHN, Division of Neurology, University of Toronto, Toronto, ON Canada; 2 Krembil Research Institute, Toronto, ON Canada; 3 Movement Disorders Unit, San Carlos Clinical Hospital, Complutense University of Madrid, Madrid, Spain; 4 Movement Disorders Unit, Neurological Institute, Tel Aviv Medical Center, Tel Aviv University, Tel Aviv-Yafo, Israel; 5 Department of Neurology and Center of Clinical Neurosciences, 1st Faculty of Medicine, Charles University in Prague and General University Hospital in Prague, Prague, Czech Republic; 6 AbbVie Inc., North Chicago, IL USA; 7 Victor Babes University of Medicine and Pharmacy, Timisoara, Romania

**Keywords:** Parkinson's disease, Long-term treatment, Dyskinesia, Motor symptoms, Nonmotor symptoms

## Abstract

**Background:**

While immediate benefits of levodopa–carbidopa intestinal gel (LCIG) are evident in patients with Parkinson’s disease (PD), long-term LCIG effects require further study.

**Objectives:**

We explored long-term LCIG on motor symptoms, nonmotor symptoms (NMS), and LCIG treatment settings in patients with advanced PD (APD).

**Methods:**

Data were obtained (medical records and patient visit) from COSMOS, a multinational, retrospective, cross-sectional post-marketing observational study in patients with APD. Patients were stratified into 5 groups based on LCIG treatment duration at the patient visit, from 1–2 to > 5 years LCIG. Between-group differences were assessed for changes from baseline in LCIG settings, motor symptoms, NMS, add-on medications, and safety.

**Results:**

Out of 387 patients, the number of patients per LCIG group was: > 1– ≤ 2 years LCIG (*n* = 156); > 2– ≤ 3 years LCIG (*n* = 80); > 3– ≤ 4 years LCIG (*n* = 61); > 4– ≤ 5 years LCIG (*n* = 30); > 5 years LCIG (*n* = 60). Baseline values were similar; data reported are changes from the baseline. There were reductions in “off” time, dyskinesia duration, and severity across LCIG groups. Prevalence, severity, and frequency of many individual motor symptoms and some NMS were reduced amongst all LCIG groups, with few differences between groups. Doses for LCIG, LEDD and LEDD for add-on medications were similar across groups both at LCIG initiation and patient visit. Adverse events were similar across all LCIG groups and consistent with the established safety profile of LCIG.

**Conclusions:**

LCIG may provide sustained, long-term symptom control, while potentially avoiding increases in add-on medication dosages.

**Trial registration:**

ClinicalTrials.gov Identifier: NCT03362879. Number and date: P16-831, November 30, 2017.

**Supplementary Information:**

The online version contains supplementary material available at 10.1007/s00415-023-11615-3.

## Introduction

Levodopa is the current gold standard therapy for the improvement of motor symptoms in patients with Parkinson’s disease (PD) [1]. However, long-term oral levodopa administration is associated with irregular, pulsatile dopamine stimulation and insufficient symptom control in patients with PD [2–7]. Progressive loss of striatal dopamine neurons and compromised dopamine signaling over time can contribute to greater motor complications in some patients with PD [2, 4]. Specifically, long-term use of oral levodopa (4–6 years) can be associated with up to a 40–75% likelihood of developing disabling motor complications and a 40% dyskinesia risk [1, 8, 9]. Further, the short levodopa half-life and irregular gastric emptying can lead to erratic plasma levodopa levels [2, 4, 10, 11]. Without consistent levodopa brain influx, patients are less likely to achieve a clinical response with oral levodopa over time [1]. Continuous dopaminergic stimulation produces several advantages over pulsatile dopaminergic stimulation [12]. Taken together, oral levodopa may not provide sufficient symptom control or clinical response as motor symptoms worsen over time in patients with PD.

Motor disturbances with long-term levodopa are also associated with a variety of nonmotor symptoms (NMS), all of which contribute to reduced health-related quality of life [1, 2, 13]. Treatment regimens include multiple medications aimed to increase dopamine neurotransmission (eg, addition of dopamine agonists) or to prolong the oral levodopa effect (eg, addition of monoamine oxidase [MAO] or catecholamine-*O*-methyltransferase [COMT] inhibitors) [14, 15]. These treatment regimens may provide symptom control for a period of time, but are often associated with drug-drug interactions, risk for medication errors, and reduced treatment adherence [14, 16, 17]. Additionally, add-on medications can have undesired side effects, such as impulse control disorders with dopamine agonists or diarrhea with COMT inhibitors [18, 19]. Likewise, medication non-adherence is prevalent in patients with PD, especially those with cognitive impairment or swallowing difficulties, leading to further motor dysfunction, as well as increased healthcare costs [1, 16, 20–24].

Levodopa–carbidopa intestinal gel (LCIG) allows individualized doses of levodopa to be infused continuously into the absorption site at the small intestine to maintain stable physiological dopamine levels [2, 25]. In clinical trials and real-world studies, LCIG leads to significantly greater improvements in PD symptom control and health-related quality of life, as compared to levodopa [2, 4, 26–31]. Likewise, a randomized, crossover design comparator study found that patients generally preferred infusion therapy with LCIG versus oral administration with levodopa (*n* = 24) [32]. Indeed, meta-analyses have found significant improvements in off-time and quality of life or comparable benefits with LCIG versus deep brain stimulation, continuous subcutaneous apomorphine infusion, and best medical treatment [33–35], and similar cost between LCIG and standard of care [36].

Studies on efficacy and safety supporting the use of LCIG vary in design, duration (3 weeks to 12 months), and patient population, with a few studies spanning over the course of years (2–5 years) [2, 4, 26–30]. More specifically, few studies have investigated the long-term use of LCIG monotherapy, with the longest durations being 2 years in the Global Long-term Registry on Efficacy and Safety of LCIG in patients with APD (GLORIA) study [37, 38], 3 years in the DUOGLOBE multinational real-world observational study [39], and in one study for up to 10 years (*n* = 37) [40]. There is a need for further clinical evidence on the long-term use of LCIG in patients with APD.

The COmedication Study assessing Mono- and cOmbination therapy with levodopa–carbidopa inteStinal gel (COSMOS) is a large, multinational study dedicated to evaluating the use of LCIG as a monotherapy or combination therapy [41]. Here, we report on long-term LCIG effects on motor symptoms, NMS, patient-reported outcomes, LCIG treatment settings, and safety according to LCIG treatment duration.

## Methods

### Study design

COSMOS was a multinational, retrospective, and cross-sectional post-marketing observational study in patients with APD treated with LCIG in routine clinical care (Clinicaltrials.gov identifier: NCT03362879; Fasano et al. [41] for full methods). Briefly, data were collected retrospectively via chart review and cross-sectionally at a single study patient visit. Clinical data were entered into a web-based electronic data capture system for analysis.

### Participants

Patients were eligible for inclusion if they had a diagnosis of APD and had received ongoing LCIG treatment for ≥ 12 months prior to the study visit. Patients were excluded if they had participated in a concurrent or prior clinical trial involving LCIG or were unable to complete the study questionnaire. Written informed consent was obtained by each patient or legally authorized representative prior to any data collection. For this analysis, patients were stratified into five groups based on LCIG treatment duration at the patient visit: 1–2 years LCIG (> 1 to ≤ 2 years LCIG), 2–3 years LCIG (> 2 to ≤ 3 years LCIG), 3–4 years LCIG (> 3 to ≤ 4 years LCIG), 4–5 years LCIG (> 4 to ≤ 5 years LCIG), and > 5 years LCIG.

### Demographics and clinical characteristics

During the patient visit, physicians collected current patient demographic information and medical history. Medical history data included PD history, clinical PD status, and LCIG treatment settings.

### Clinical assessments

At baseline, defined as immediately prior to LCIG initiation and at the patient visit, physicians used the Unified Parkinson’s Disease Rating Scale (UPDRS) to measure “off” time (Part IV item 39, modified) and dyskinesia duration (Part IV item 32, modified), as well as dyskinesia severity (Part IV item 33). The UPDRS was selected based on the feasibility of completion within the clinical practice setting. “Off” time and dyskinesia duration were documented as hours during the day prior to the clinical visit as reported by the patient. At the patient visit, physicians assessed the prevalence, severity rating (none, mild, moderate, or severe), and frequency rating (rarely, often, frequent, or very frequent) of motor symptoms, NMS, and symptoms related to treatment from both timepoints (at baseline and at the patient visit). Symptoms were defined as characterized by the PD composite scale [42]. In addition, patients were assessed with the NMS Scale (NMSS), PD Sleep Scale Version 2 (PDSS-2), and PD Quality of Life Questionnaire (PDQ-8) at the patient visit. LCIG dosing and infusion-related dosing parameters were collected from medical records and at the patient visit. Data on add-on medications were collected.

### Safety

Data from safety assessments were previously collected by healthcare professionals and documented in medical records along with any prior suspected adverse reactions from the patients’ medical files. Adverse events (AEs) possibly related to treatment or device were documented.

### Statistical analysis

Data from medical records and patient visits were analyzed using descriptive statistics. All statistical analyses were carried out using SAS^®^ version 9.4 (SAS Institute, Inc., Cary, NC, USA). Prevalence, defined as the percentage of patients affected, was analyzed for motor symptoms, NMS, and symptoms related to treatment. For motor symptoms, NMS, and treatment-related symptoms, ratings of frequency and severity were transformed into numerical scores and analyzed. For each symptom, variables ‘severity’ and ‘frequency’ were transformed as follows: for the transformed severity variable 'no symptom' was 0, mild = 1, moderate = 2, and severe = 3, and unknown was set to missing. For the transformed frequency variable 'no symptom' was 0, rarely = 1, often = 2, frequent = 3, very frequent = 4, and unknown was set to missing. A chi-squared test was used to compare categorical data (eg, PD motor phenotype, monotherapy, and motor symptom and NMS prevalence when possible). The Kappa test was used to analyze motor symptoms and NMS prevalence (within-group differences). The Wilcoxon test was used to analyze quantitative comparisons of continuous, non-normally distributed data (eg, between-group and within-group differences of severity and frequency scores for NMSS, PDSS-2, and PDQ-8).

## Results

### Participants

The COSMOS study included 409 patients from 49 sites in 14 countries [41]. A total of 387 patients were included in this retrospective analysis based on data availability from medical records at the patient visit date (ie, 22 patients were omitted due to lack of data on the LCIG initiation date in case report forms and the inability to calculate LCIG duration). The number of patients per LCIG duration were as follows: 1–2 years LCIG (*n* = 156); 2–3 years LCIG (*n* = 80); 3–4 years LCIG (*n* = 61); 4–5 years LCIG (*n* = 30); > 5 years LCIG (*n* = 60). Demographics and clinical characteristics at baseline were generally similar among groups (Table 1). The majority of patients were male with a mean ± standard deviation (SD) age of patients at baseline ranging from 64.5 ± 7.6 years of age (4–5 years LCIG) to 67.9 ± 7.4 years of age (1–2 years LCIG) (Table 1). Across all groups, the most common reason for LCIG initiation was due to disabling motor fluctuations/off periods (≥ 83.3%) (Table 1). The other most common reasons for LCIG initiation were decreased quality of life, uncontrolled dyskinesia, and lack of efficacy of previous treatment. The time from PD diagnosis to LCIG initiation ranged from a mean ± SD of 12.1 ± 5.4 years (1–2 years LCIG) to 13.8 ± 5.1 years (> 5 years LCIG) (Table 1). At baseline, patients in the 4–5 years LCIG group had lower mean ± SD dyskinesia duration (2.7 ± 2.6 h) as compared to other groups, ranging from mean ± SD of 3.1 ± 2.9 to 4.9 ± 4.5 h (Table 1).

**Table 1:** Baseline demographics and clinical characteristics of patients according to duration of LCIG treatment

Characteristic	Time between LCIG initiation and patient visit				
	1–2 years *n* = 156	2–3 years *n* = 80	3–4 years *n* = 61	4–5 years *n* = 30	> 5 years *n* = 60
Duration of LCIG treatment, months, mean ± SD	16.6 ± 3.9	30.3 ± 3.3	41.8 ± 3.7	53.7 ± 3.3	78.8 ± 18.6
Male, *n* (%)	100 (64.1%)	54 (67.5%)	37 (60.7%)	19 (63.3%)	42 (70.0%)
Age at LCIG initiation, years, mean ± SD	67.9 ± 7.4	66.2 ± 8.4	65.8 ± 7.5	64.5 ± 7.6	65.0 ± 8.2
Race, *n* (%)^a^					
White	153 (98.1)	80 (100)	61 (100)	30 (100)	60 (100)
Asian	1 (0.6)	0	0	0	0
Other	2 (1.3)	0	0	0	0
Disease history					
PD motor phenotype, *n* (%)					
Akinetic rigid	61 (39.1)	29 (36.3)	26 (42.6)	11 (37.9)	22 (36.7)
Mixed	45 (28.8)	31 (38.8)	18 (29.5)	6 (20.7)	20 (33.3)
Tremor-dominant	46 (29.5)	19 (23.8)	16 (26.2)	11 (37.9)	17 (28.3)
Unknown	2 (1.3)	1 (1.3)	1 (1.6)	0	1 (1.7)
Other	2 (1.3)	0	0	1 (3.4)^b^	0
Morning akinesia present, *n* (%)	110 (71.0)	51 (65.4)	42 (70.0)	22 (78.6)	41 (71.9)
Wearing off present, *n* (%)	147 (94.2)	73 (93.6)	61 (100.0)	27 (93.1)	54 (91.5)
“Off” time, hours, mean ± SD, (*n*)	5.8 ± 3.7 (112)	5.7 ± 2.6 (56)	6.4 ± 3.9 (44)	5.8 ± 3.9 (22)	7.2 ± 3.8 (35)
Dyskinesia present, *n* (%)	129 (82.7)	60 (76.9)	54 (88.5)	22 (75.9)	55 (91.7)
Dyskinesia duration, hours, mean ± SD, (*n*)	3.1 ± 2.9 (113)	4.9 ± 4.5 (51)	4.0 ± 3.0 (43)	2.7 ± 2.6 (21)	4.9 ± 3.4 (33)
Dyskinesia severity, mean ± SD (*n*)	1.5 ± 1.1 (144)	1.5 ± 1.2 (72)	1.8 ± 1.2 (59)	1.5 ± 1.1 (29)	1.8 ± 1.1 (51)
Time from PD diagnosis to LCIG initiation, years, mean ± SD (*n*)	12.1 ± 5.4 (156)	13.0 ± 5.4 (80)	13.7 ± 5.4 (61)	12.2 ± 5.7 (30)	13.8 ± 5.1 (60)
Time from PD diagnosis to wearing off^c^, years, mean ± SD (*n*)	6.8 ± 3.6 (143)	8.1 ± 4.3 (69)	8.3 ± 3.7 (60)	7.6 ± 4.3 (27)	8.3 ± 4.3 (53)
Time from PD diagnosis to dyskinesia^c^, years, mean ± SD (*n*)	7.7 ± 4.2 (124)	8.4 ± 3.6 (57)	8.8 ± 3.6 (53)	9.1 ± 5.3 (22)	9.4 ± 4.3 (55)
Reason for LCIG initiation, *n* (%)^d^					
Disabling motor fluctuations/off periods	138 (88.5)	72 (90.0)	60 (98.4)	25 (83.3)	58 (96.7)
Decreased quality of life	85 (54.5)	46 (57.5)	34 (55.7)	19 (63.3)	41 (68.3)
Uncontrolled dyskinesia	77 (49.4)	36 (45.0)	36 (59.0)	17 (56.7)	36 (60.0)
Lack of efficacy of previous treatment	76 (48.7)	49 (61.3)	33 (54.1)	12 (40.0)	32 (53.3)
Safety	16 (10.3)	10 (12.5)	8 (13.1)	4 (13.3)	9 (15.0)
Other	1 (0.6)	3 (3.8)	0 (0)	2 (6.7)	4 (6.7)

*LCIG* levodopa–carbidopa intestinal gel, *PD* Parkinson’s disease, *SD* standard deviation

^a^There were no patients who were black or mixed race

^b^There was also one missing case

^c^Restricted to patients with respective diagnosis

^d^Multiple answers possible

### Clinical assessments

#### “Off” time, “on” time, and dyskinesia severity

**Fig. 1:**
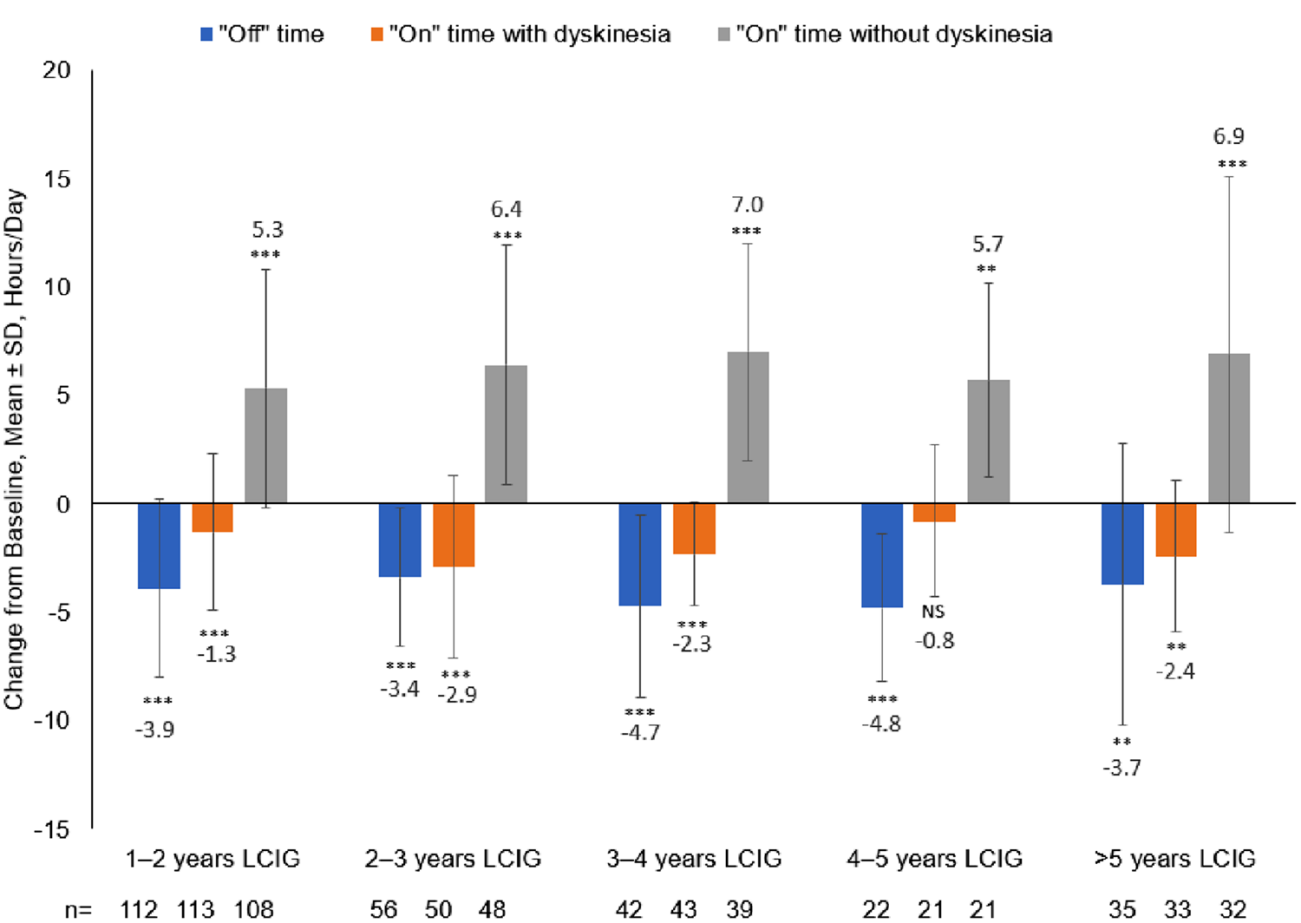
Change from Baseline of “Off” Time and “On” Time with and without Dyskinesia During Waking Hours from LCIG Initiation to Patient Visit According to Duration of LCIG Treatment. Significance for change from baseline as follows: NS: *p* > 0.05; **p < 0.001; ***p < 0.0001. *LCIG* levodopa–carbidopa intestinal gel, *NS* not significant, *SD* standard deviation

At the time of the patient visit, the duration of “off” time was reduced from baseline in all groups (*p* < 0.001) (Fig. 1). The duration of “on” time with dyskinesia was reduced from baseline in all (*p* < 0.001), with the exception of the 4–5 years LCIG group (*p* = 0.1378). “On” time without dyskinesia was increased from baseline in all groups (*p* < 0.0001 for all groups except 4–5 years LCIG [*p* = 0.0002]) (Fig. 1). Reductions from baseline were found in dyskinesia severity: mean ± SD, ranging from − 0.7 ± 1.3 to − 0.9 ± 1.3 (*p* < 0.0001 for all groups except 4–5 years LCIG [*p* = 0.0284] and > 5 years LCIG [*p* = 0.0003]).

### Motor symptoms, NMS, and treatment-related symptoms

**Fig. 2:**
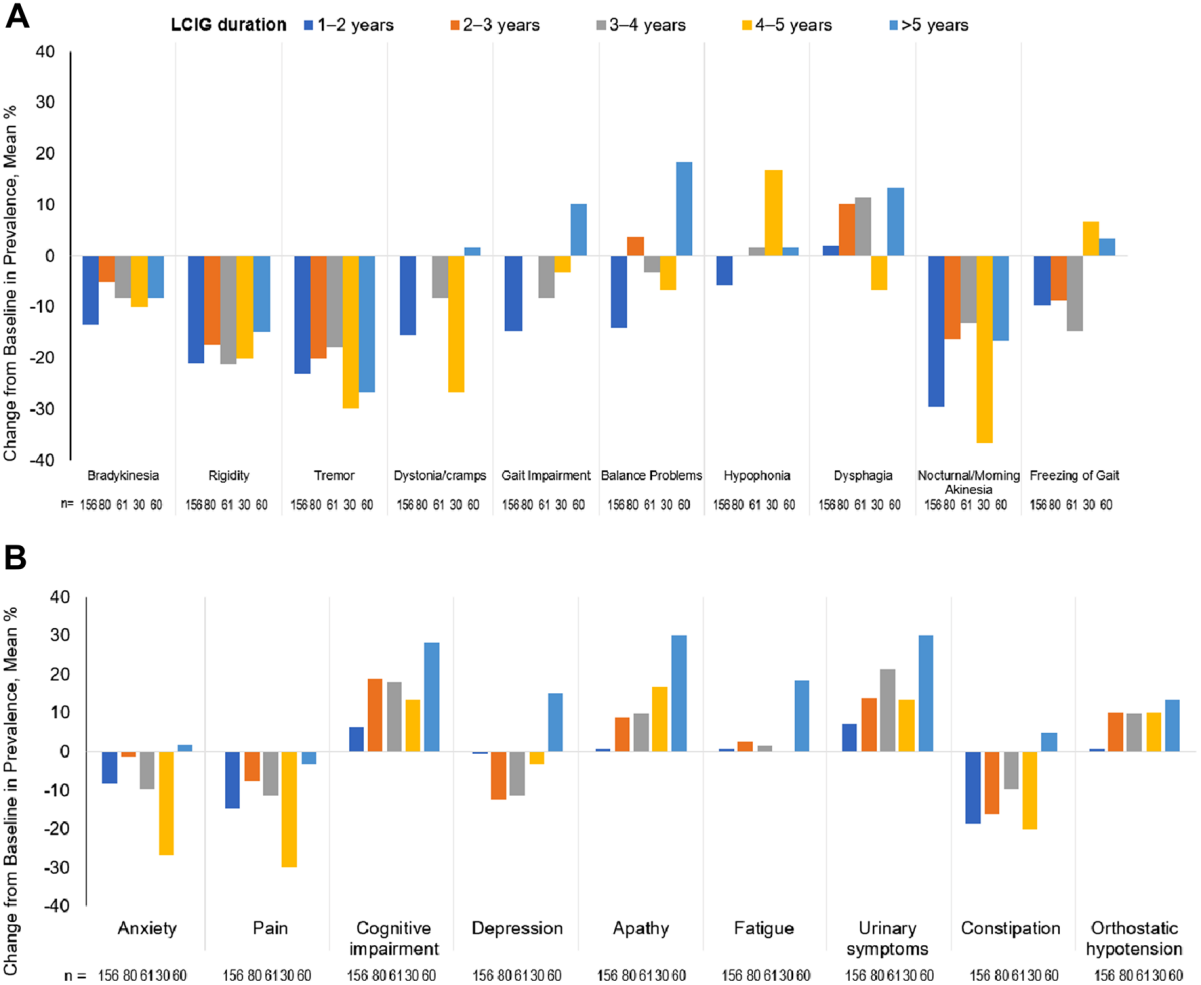
Change from Baseline in Prevalence of Motor Symptoms (**A**) and NMS (**B**). According to Duration of LCIG Treatment. Negative values indicate a decrease in symptom prevalence. *LCIG* levodopa–carbidopa intestinal gel, *NMS* non-motor symptoms

For many of the individual motor symptoms, reductions from baseline in prevalence were found in all groups for tremor and nocturnal/morning akinesia, while dysphagia increased (all groups except 4–5 years LCIG) and hypophonia increased (4–5 years LCIG only) (Fig. 2A). Some individual NMS showed similar reductions from baseline in all groups for the prevalence of anxiety, pain, constipation, gambling, and dopamine dysregulation syndrome, with the exception of constipation (> 5 years LCIG) and dopamine dysregulation syndrome (1–2 years LCIG and > 5 years LCIG) (Fig. 2B). There were no significant changes from baseline in the prevalence of treatment-related symptoms (Supplemental Fig. 1).

Reductions from baseline in severity and frequency were seen in individual motor symptoms, specifically for bradykinesia, rigidity, tremor, dystonia/cramps, gait impairment, nocturnal/morning akinesia, and freezing of gait for all groups (Supplemental Figs. 2A and 3A). Similarly, patients in all groups had reductions from baseline in the severity and frequency of some of the individual NMS and treatment-related symptoms assessed (Supplemental Figs. 2B, C and 3B, C). Differences between groups are noted in Supplemental Figs. 2 and 3.

### NMSS, PDSS-2, and PDQ-8

NMSS scores were lower with 1–2 and 2–3 years LCIG vs > 5 years LCIG (mean ± SD, 1–2 years LCIG [55.3 ± 43.8]; 2–3 years LCIG [52.6 ± 38.3] vs > 5 years LCIG [68.9 ± 37.4], *p* = 0.0068 and *p* = 0.0057) (Table 2). PDSS-2 total scores were similar across groups at the patient visit (mean ± SD, ranging from 18.9 ± 9.1 to 22.0 ± 11.9) (Table 2)*.* PDQ-8 was lower with 1–2 years and 2–3 years LCIG as compared to > 5 years LCIG (mean ± SD, 1–2 years LCIG [36.5 ± 19.0] and 2–3 years LCIG [38.9 ± 18.5] vs > 5 years LCIG [46.3 ± 16.5]; *p* = 0.0003 and *p* = 0.0076) (Table 2). Differences between groups with *p* < 0.05 are as follows: NMSS, 1–2 years vs > 5 years (*p* = 0.0068) and 2–3 years vs > 5 years (*p* = 0.0057); PDQ-8, 1–2 years vs 3–4 years (*p* = 0.0052), 1–2 years vs > 5 years (*p* = 0.0003), 2–3 years vs > 5 years (*p* = 0.0076). No differences between groups were found in PDSS-2.

**Table 2:** NMSS, PDSS, and PDQ-8 scores assessed via questionnaires according to duration of LCIG treatment

Measure	Time between LCIG initiation and patient visit									
	1–2 years *n* = 156		2–3 years *n* = 80		3–4 years *n* = 61		4–5 years *n* = 30		> 5 years *n* = 60	
	*n*	Mean ± SD	*n*	Mean ± SD	*n*	Mean ± SD	*n*	Mean ± SD	*n*	Mean ± SD
NMSS total score	155	55.3 ± 43.8	79	52.6 ± 38.3	61	65.7 ± 46.2	29	62.9 ± 43.4	59	68.9 ± 37.4
PDSS-2 total score	148	19.7 ± 10.6	74	20.2 ± 10.8	59	21.9 ± 10.4	26	18.9 ± 9.1	56	22.0 ± 11.9
PDQ-8 summary index	154	36.5 ± 19.0	79	38.9 ± 18.5	60	43.9 ± 15.6	30	40.6 ± 14.3	59	46.3 ± 16.5

*LCIG* levodopa–carbidopa intestinal gel, *NMSS* Non-Motor Symptoms Scale, *PDSS-2* Parkinson’s Disease Sleep Scale, *PDQ* Parkinson’s Disease Questionnaire, *SD* standard deviation

### LCIG dosage and add-on medications

**Fig. 3:**
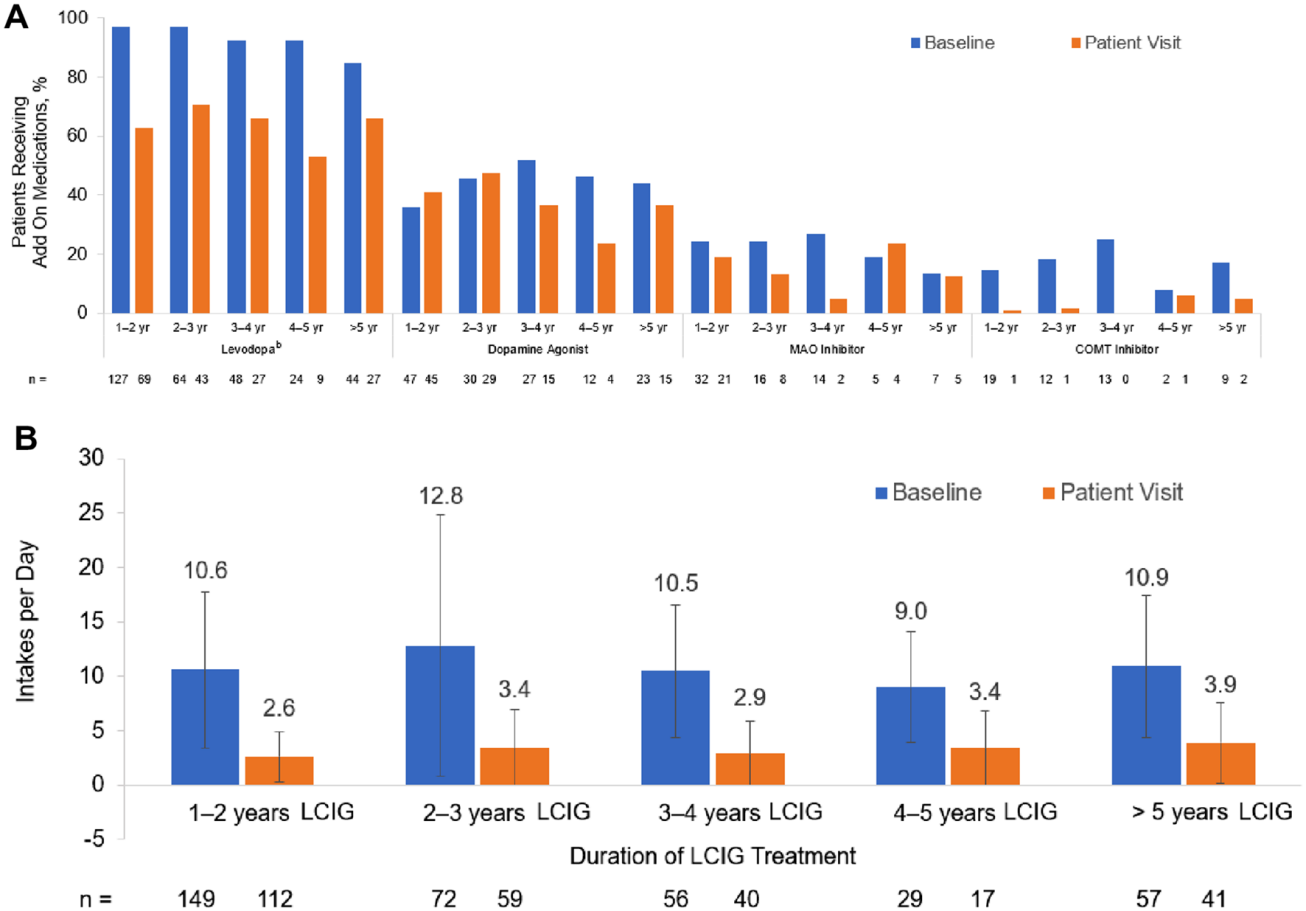
Percentage of Patients Receiving Add-on Medications (**A**) and Add-on PD Medication Intakes (**B**) at Baseline and Patient Visit^a^. ^a^Less than 10% of patients had any intake of apomorphine, anticholinergics, and other add-on medications (not shown). No changes were found with NMDA antagonists as add-on medications (not shown). ^b^Levodopa treatment included nocturnal levodopa, among other reasons, at the patient visit. *COMT* catecholamine-*O*-methyltransferase, *LCIG* levodopa–carbidopa intestinal gel, *MAO* monoamine oxidase, *NMDA N*-methyl-d-aspartate, *PD* Parkinson’s disease

LCIG total doses and LEDD were similar across groups at both LCIG initiation and at the patient visit (Table 3). Add-on medications were reduced in most groups at the patient visit as compared to baseline (Fig. 3A). Additionally, the number of add-on medication intakes were reduced at the patient visit as compared to baseline, independent of LCIG duration (*p* < 0.0001) (Fig. 3B). At 12 months after LCIG initiation, the percentages of patients receiving LCIG as monotherapy ranged from 22.4 to 46.7%, while the percentages of those receiving LCIG monotherapy plus night medication ranged from 10.0 to 30.3% and those receiving polytherapy ranged from 40.1 to 52.6% (Table 3).

**Table 3:** LCIG settings and LEDD at baseline and at the patient visit according to duration of LCIG treatment

Measure	Time point	Time between LCIG initiation and patient visit									
		1–2 years *n* = 156		2–3 years *n* = 80		3–4 years *n* = 61		4–5 years *n* = 30		> 5 years *n* = 60	
		*n*	Mean ± SD	*n*	Mean ± SD	*n*	Mean ± SD	*n*	Mean ± SD	*n*	Mean ± SD
Total LCIG dose (mg)	At LCIG initiation	155	1274.4 ± 524.9	80	1326.1 ± 480.0	60	1307.0 ± 487.7	30	1314.6 ± 628.7	60	1389.4 ± 392.7
	At patient visit	154	1409.8 ± 547.9	78	1388.8 ± 560.7	60	1385.6 ± 509.3	30	1446.4 ± 769.5	60	1480.9 ± 666.1
LEDD (mg)	At LCIG initiation	133	2422.3 ± 1033.8	64	2659.1 ± 1003.9	50	2647.5 ± 1011.6	27	2236.8 ± 924.6	50	2585.5 ± 1034.8
	At patient visit	146	2162.4 ± 842.2	75	2225.7 ± 858.8	57	2166.5 ± 850.3	29	2199.3 ± 1098.8	55	2401.2 ± 1092.1

		*n*	%	*n*	%	*n*	%	*n*	%	*n*	%
Patients on LCIG monotherapy, %	At 12 months after LCIG initiation	45	29.6	17	22.4	19	32.2	14	46.7	23	39.7
Patients on LCIG monotherapy plus night medication, %	At 12 months after LCIG initiation	46	30.3	19	25.0	15	25.4	3	10.0	10	17.2
Patients on polytherapy, %	At 12 months after LCIG initiation	61	40.1	40	52.6	25	42.4	13	43.3	25	43.1

*LCIG* levodopa–carbidopa intestinal gel, *LEDD* levodopa equivalent daily dose, *SD* standard deviation

### Safety

A total of 109 AEs of any type were recorded via medical records during LCIG initiation and LCIG maintenance treatment for 387 patients, ranging across groups from 24% (37/156) in 1–2 years LCIG to 36% (22/61) in 3–4 years LCIG. The most common AEs reported were stoma site infection (> 5 years LCIG: 4/60 [7%] and 3–4 years LCIG: 3/61 [5%]) and unintentional medical device removal (3–4 years LCIG: 3/61 [5%]).

## Discussion

In this post hoc analysis of the retrospective, cross-sectional, multinational COSMOS study, we reported LCIG treatment benefits present in the short-term and sustained through long-term LCIG treatment. Motor fluctuations, “off” time, dyskinesia, and motor symptoms improved from baseline for all groups, independent of LCIG duration. With continuous levodopa infusion and subsequent continuous dopaminergic stimulation, patients were able to maintain symptom control beyond 5 years with a stable levodopa requirement while reducing the burden of a complex add-on medication regimen. Approximately 20–45% of patients were on LCIG monotherapy at least 12 months after LCIG initiation, with 39.7% receiving LCIG monotherapy (and another 17.2% receiving LCIG monotherapy plus nighttime medication) after more than 5 years of treatment. The improvement of dyskinesia duration and severity, as well as “off” time, and the large percentage of patients on LCIG monotherapy, as found in the COSMOS primary analysis [41], support the potential use of LCIG for long-term symptom control.

A major strength of this study was the use of real-world, multinational, large-cohort clinical data. This post-hoc analysis of the COSMOS study is one of the few analyses to evaluate long-term LCIG therapy in patients with APD, and the first to systematically compare outcomes according to treatment duration, as well as long-term data for the rate of monotherapy, LEDD, and a number of intakes. The improvements in motor symptoms and NMS are supported by previous observational studies, as well as randomized controlled trials that have identified improvements in motor complications and NMS associated with LCIG treatment [2, 4, 26–30, 38].

The study included a partially retrospective design and observational analyses, without randomization at the study start, which limited data collection and interpretation. In addition, this study included only patients treated with ongoing LCIG who were able to sustain LCIG treatment for at least 12 months. Thus, the results of this analysis are not representative of all patients who initiate LCIG, possibly introducing an unintended bias. However, given the nature of the study, it is conceivable that patients with suboptimal outcomes over time discontinued LCIG and were not included in the analysis. In addition, recall bias at the patient visit may also influence the data upon which this post hoc analysis is based, wherein events that happened long ago are more likely to be misreported and thus potentially introducing systematic error across the treatment duration groups. Finally, the study was unable to achieve extended, long-term follow-up much beyond 5 years due to the low number of patients tracked for longer durations. However, this study design allows for a larger patient cohort than one would be able to track in a long-term treatment prospective design.

The data from this real-world analysis of patients with APD demonstrate patient outcome profiles with long-term LCIG treatment. Treatment with LCIG was associated with a sustained improvement in both dyskinesia duration and severity, independent of LCIG treatment duration. Stable levodopa delivery via LCIG reduced add-on medications. These results are in line with the EAN MDS guidelines for invasive therapies which identified improved time and quality of life in patients with LCIG versus oral therapy and shed light on potential patient selection for long-term LCIG treatment [43]. LCIG may be a long-term solution for carefully selected patients based on patient preferences and clinical characteristics (e.g., those without dementia) such that the benefits outweigh any potential risks [34, 44, 45]. Due to the long-term progressive nature of PD, as well as the aging process and potential treatment risks, patients with APD receiving treatments like LCIG may require consistent monitoring for worsening symptoms related to cognitive decline, vitamin B12 deficiency, or polyneuropathy [46, 47].

The study provides evidence that supports LCIG as an option to maintain adequate symptom control in the long term, despite the continued progression of PD. Adverse events were consistent with the known LCIG safety profile [2, 26, 41, 48]. Furthermore, LCIG can be a long-term solution for patients with memory or swallowing difficulties associated with APD who follow multi-faceted therapeutic regimens requiring many pills per day and may even eliminate the need to use add-on medications, as monotherapy was an option for many patients. Full understanding of the potential long-term benefits, beyond symptom control, with continuous dopaminergic stimulation via continuous levodopa delivery will require additional studies. In conclusion, long-term use of LCIG maintains reductions in “off” time, dyskinesia duration and severity, reduces the burden of several motor symptoms and NMS, and lessens the need for add-on medications, with a stable requirement of LCIG.

## Supplementary Information

Below is the link to the electronic supplementary material.

Supplementary file1 (DOCX 342 KB)

## Data Availability

AbbVie is committed to responsible data sharing regarding the clinical trials we sponsor. This includes access to anonymized, individual, and trial-level data (analysis data sets), as well as other information (eg, protocols and Clinical Study Reports), as long as the trials are not part of an ongoing or planned regulatory submission. This includes requests for clinical trial data for unlicensed products and indications. This clinical trial data can be requested by any qualified researchers who engage in rigorous, independent scientific research, and will be provided following review and approval of a research proposal and Statistical Analysis Plan (SAP) and execution of a Data Sharing Agreement (DSA). Data requests can be submitted at any time and the data will be accessible for 12 months, with possible extensions considered. For more information on the process, or to submit a request, visit the following link: https://www.abbvie.com/our-science/clinical-trials/clinical-trials-data-andinformation-sharing/data-and-information-sharing-with-qualified-researchers.html.
