# Supplementary material for: Levodopa–carbidopa intestinal gel in advanced Parkinson’s disease: long-term results from COSMOS
Source: J Neurol. 2023 Feb 18;270(5):2765–75. doi: 10.1007/s00415-023-11615-3 (PMC10130122; doi:10.1007/s00415-023-11615-3)
Supplement: Supplementary file 1 — Supplementary file1 (DOCX 342 KB) [file 415_2023_11615_MOESM1_ESM.docx]

Levodopa-Carbidopa Intestinal Gel in Advanced Parkinson’s Disease: Long-term Results from COSMOS

Alfonso Fasano^1,2^, Rocío García-Ramos^3^, Tanya Gurevich^4^, Robert Jech^5^, Lars Bergmann^6^, Olga Sanchez-Soliño^6^, Juan Carlos Parra^6^, Mihaela Simu^7^

^1^Edmond J Safra Program in Parkinson’s Disease and the Morton and Gloria Shulman Movement Disorders Clinic, Toronto Western Hospital – UHN, Division of Neurology, University of Toronto, Toronto, Ontario, Canada

^2^Krembil Research Institute, Toronto, Ontario, Canada

^3^Movement Disorders Unit, San Carlos Clinical Hospital, Complutense University of Madrid, Madrid, Spain

^4^**Movement Disorders Unit, Neurological Institute, Tel Aviv Medical Center, Tel Aviv University, Israel**

**^5^Department of Neurology and Center of Clinical Neurosciences, 1st Faculty of Medicine, Charles University in Prague and General University Hospital in Prague, Prague, Czech Republic**

**^6^AbbVie Inc., North Chicago, IL, USA**

^7^Victor Babes University of Medicine and Pharmacy, Timisoara, Romania

**Corresponding author:**

Alfonso Fasano

Edmond J. Safra Program in Parkinson's Disease, Morton and Gloria Shulman Movement Disorders Clinic, Toronto Western Hospital – UHN, Division of Neurology,
University of Toronto, Toronto, Ontario, Canada

Krembil Research Institute, Toronto, Ontario, Canada

Email: alfonso.fasano@uhn.ca

# Supplemental Figure 1. Change from Baseline in Prevalence of Treatment-related Symptoms According to Duration of LCIG Treatment


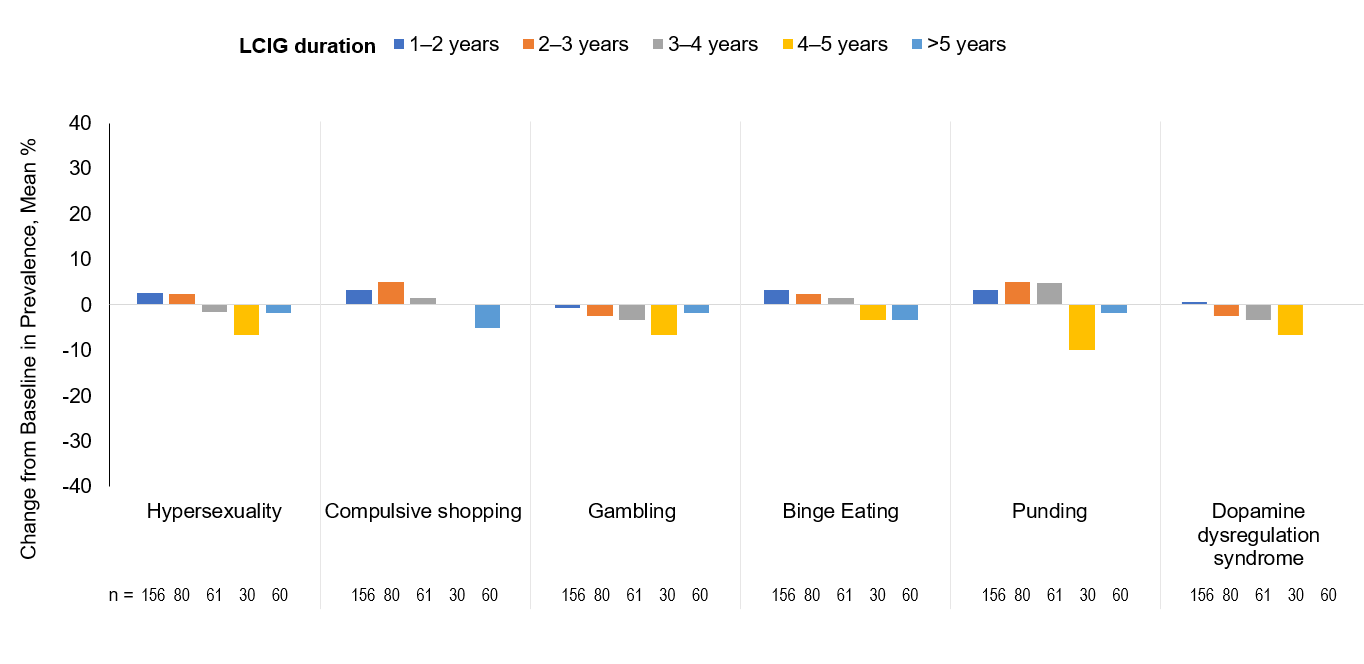


Negative values indicate a decrease in symptom prevalence.

LCIG, levodopa-carbidopa intestinal gel.

# Supplemental Figure 2. Change from Baseline in Severity of Motor Symptoms (A), NMS (B), and Treatment-related Symptoms (C) According to Duration of LCIG Treatment According to Duration of LCIG Treatment


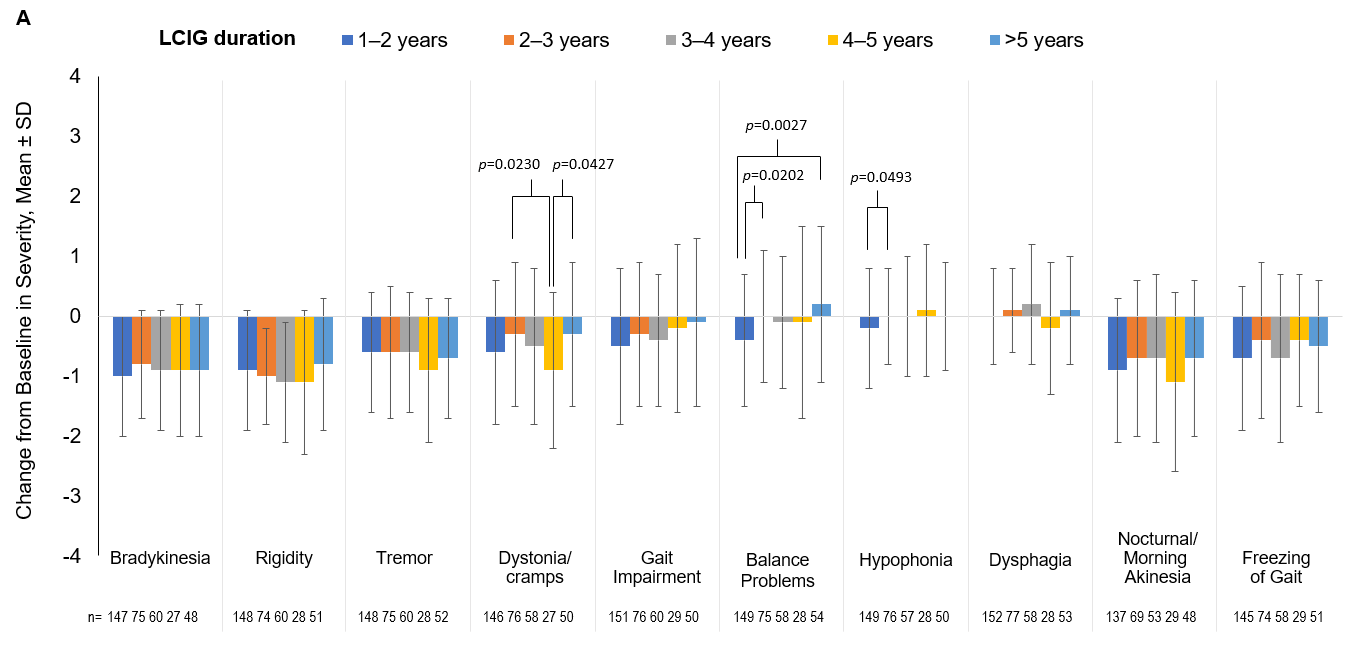


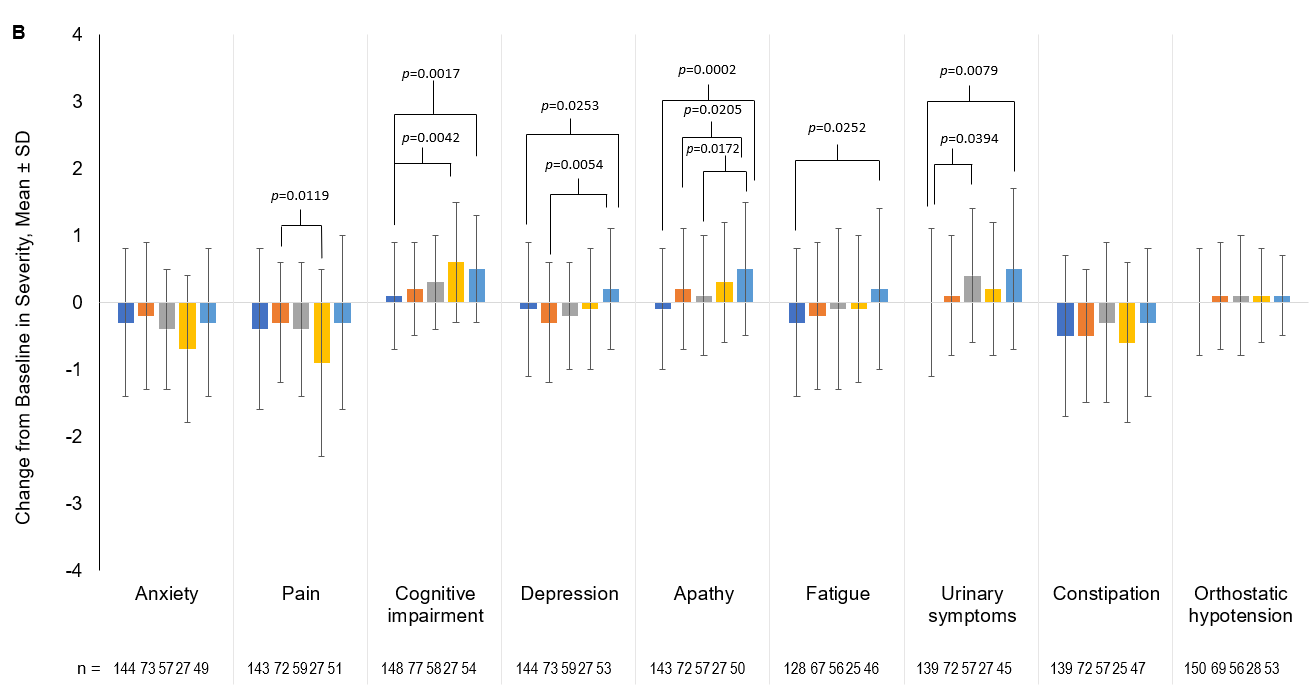


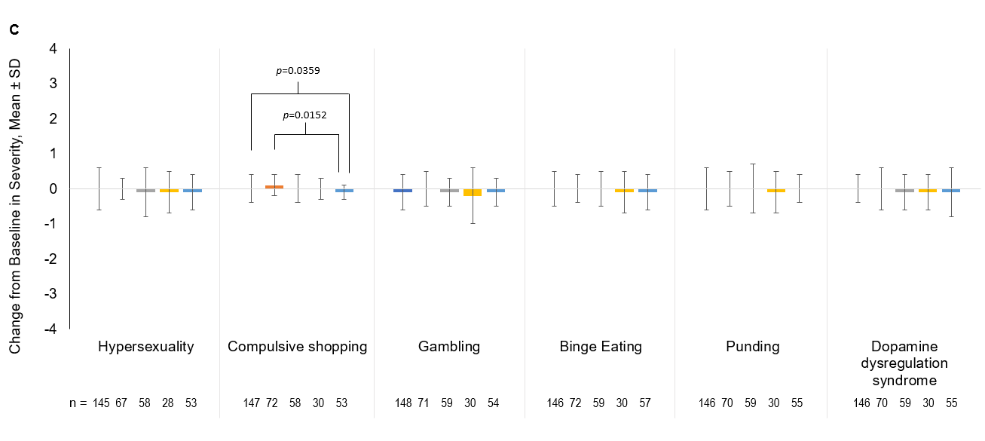


Negative values indicate a decrease in symptom severity from baseline to study visit.

LCIG, levodopa-carbidopa intestinal gel; NMS, nonmotor symptoms; SD, standard deviation.

# Supplemental Figure 3. Change from Baseline in Frequency of Motor Symptoms (A), NMS (B), and Treatment-related Symptoms (C) According to Duration of LCIG Treatment


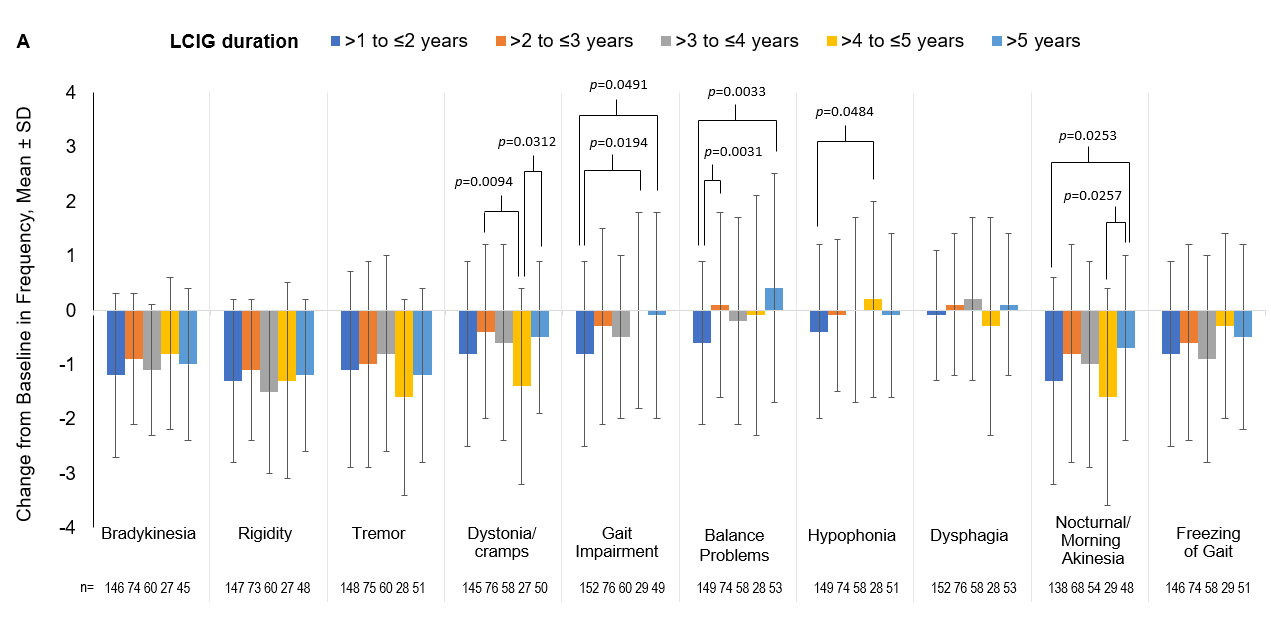


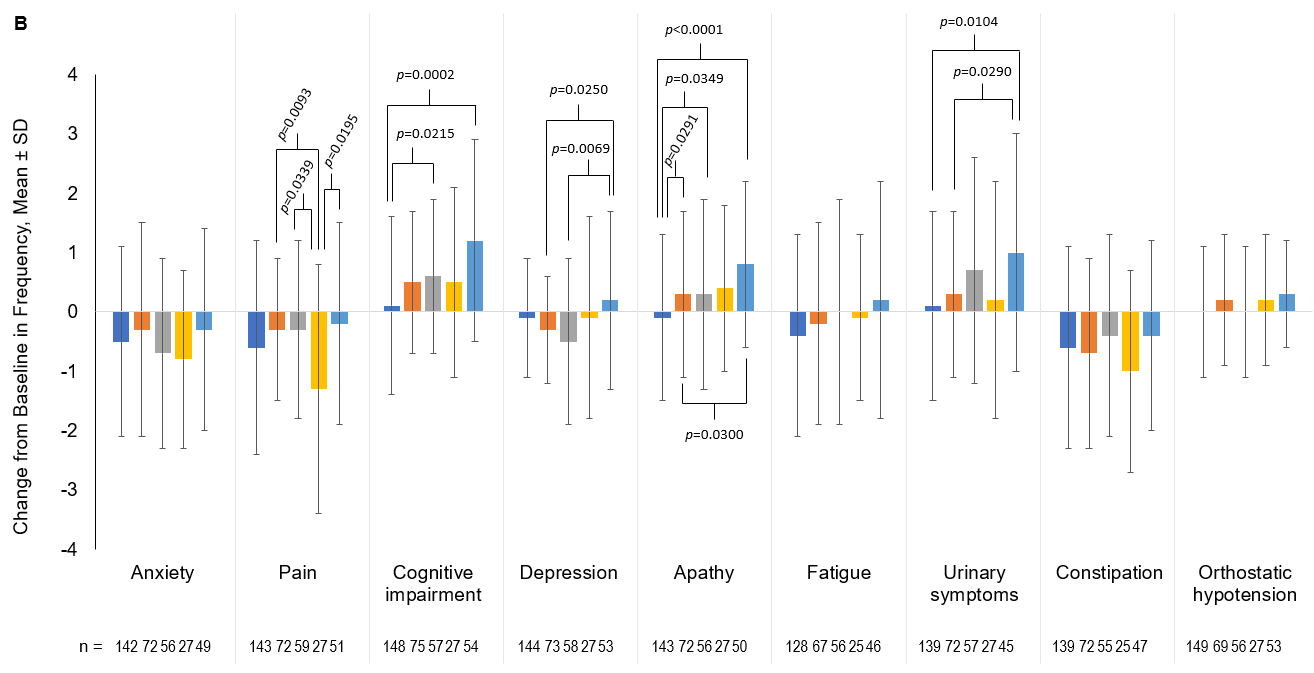


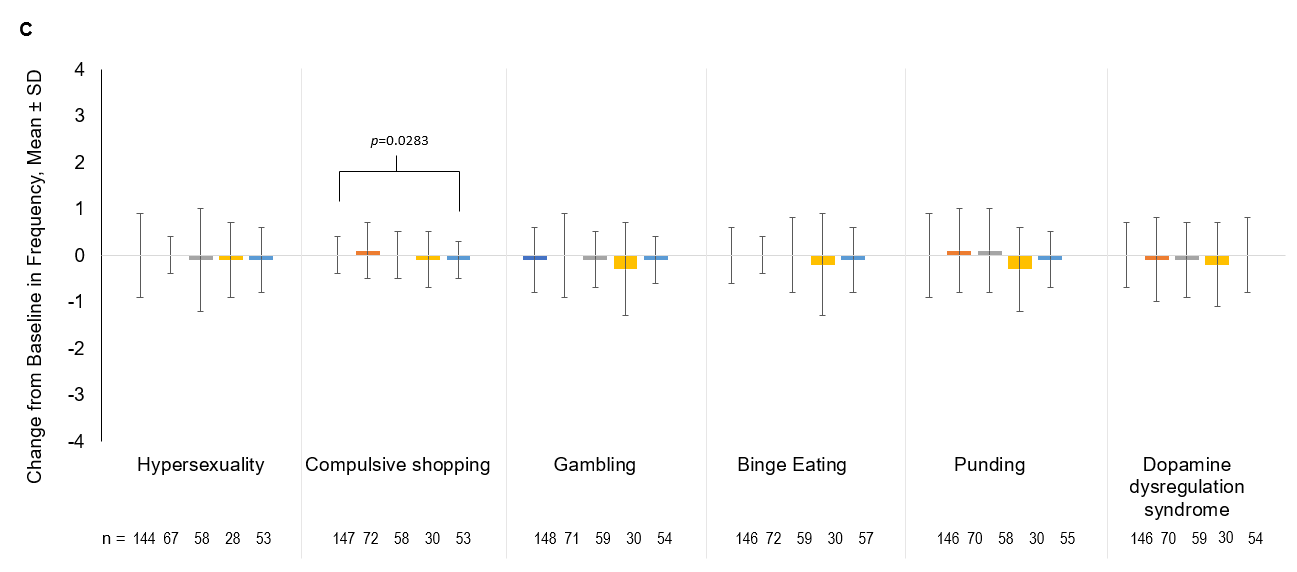


Negative values indicate a decrease in symptom frequency from baseline to study visit.

LCIG, levodopa-carbidopa intestinal gel; NMS, nonmotor symptoms; SD, standard deviation.
